# Cost-effectiveness of community-based screening and treatment of moderate acute malnutrition in Mali

**DOI:** 10.1136/bmjgh-2018-001227

**Published:** 2019-04-28

**Authors:** Sheila Isanaka, Dale A Barnhart, Christine M McDonald, Robert S Ackatia-Armah, Roland Kupka, Seydou Doumbia, Kenneth H Brown, Nicolas A Menzies

**Affiliations:** 1 Department of Nutrition, Harvard T.H. Chan School of Public Health, Boston, Massachusetts, USA; 2 Epidemiology, Harvard T.H. Chan School of Public Health, Boston, Massachusetts, USA; 3 Children’s Hospital Oakland Research Institute, Oakland, California, USA; 4 Department of Nutrition and Program in International and Community Nutrition, University of California, Davis, CA, USA; 5 United Nations Children’s Fund, Nutrition Section, New York, NY, USA; 6 Faculty of Medicine and Odontostomatology, University of Sciences, Techniques and Technology of Bamako, Bamako, Mali; 7 Global Health and Population, Harvard T.H. Chan School of Public Health, Boston, Massachusetts, USA

**Keywords:** moderate acute malnutrition, corn soy blend, CSB++, Super Cereal, ready to use supplementary foods, PlumpySup, cost, cost-effectiveness, Mali

## Abstract

**Introduction:**

Moderate acute malnutrition (MAM) causes substantial child morbidity and mortality, accounting for 4.4% of deaths and 6.0% of disability-adjusted life years (DALY) lost among children under 5 each year. There is growing consensus on the need to provide appropriate treatment of MAM, both to reduce associated morbidity and mortality and to halt its progression to severe acute malnutrition. We estimated health outcomes, costs and cost-effectiveness of four dietary supplements for MAM treatment in children 6–35 months of age in Mali.

**Methods:**

We conducted a cluster-randomised MAM treatment trial to describe nutritional outcomes of four dietary supplements for the management of MAM: ready-to-use supplementary foods (RUSF; PlumpySup); a specially formulated corn–soy blend (CSB) containing dehulled soybean flour, maize flour, dried skimmed milk, soy oil and a micronutrient pre-mix (CSB++; Super Cereal Plus); Misola, a locally produced, micronutrient-fortified, cereal–legume blend (MI); and locally milled flour (LMF), a mixture of millet, beans, oil and sugar, with a separate micronutrient powder. We used a decision tree model to estimate long-term outcomes and calculated incremental cost-effectiveness ratios (ICERs) comparing the health and economic outcomes of each strategy.

**Results:**

Compared to no MAM treatment, MAM treatment with RUSF, CSB++, MI and LMF reduced the risk of death by 15.4%, 12.7%, 11.9% and 10.3%, respectively. The ICER was US$9821 per death averted (2015 USD) and US$347 per DALY averted for RUSF compared with no MAM treatment.

**Conclusion:**

MAM treatment with RUSF is cost-effective across a wide range of willingness-to-pay thresholds.

**Trial registration:**

NCT01015950.

**Key questions**

**What is already known?**

- Given the large burden of moderate acute malnutrition (MAM, 34 million children affected each year) and risk of progression to more life-threatening conditions without adequate treatment, the management of MAM should be considered a public health priority.
- The cost and cost-effectiveness of MAM treatment with new dietary supplements has not been assessed, but such evidence is needed to inform resource allocation for global child survival interventions.

**What are the new findings?**

- Using data from a cluster-randomised trial and a decision tree model, we estimated the long-term outcomes and incremental cost-effectiveness ratios comparing the health and economic outcomes of four dietary supplements.
- Our results show that providing MAM treatment is cost-effective across a wide range of cost-effectiveness thresholds, and that despite having the highest per-unit food costs, the provision of ready-to-use supplementary food was the optimal dietary supplement for MAM treatment, when compared with three other supplementary food options.

**What do the new findings imply?**

- MAM treatment could reduce the number of child deaths by 187 000 each year, and in settings with available resources, MAM treatment should be considered a cost-effective extension of existing child survival interventions.

## Introduction

It is estimated from survey data that 34 million children <5 years of age suffer from moderate acute malnutrition (MAM) and an additional 16 million children suffer from severe acute malnutrition (SAM).^1^ The risk of associated morbidity and mortality increases with the severity of malnutrition, but both MAM and SAM impair immune function, increase the incidence and duration of common childhood infections, and heighten the risk of mortality.^2^ Compared with non-malnourished children, the rate of death is 3 times greater among children with MAM and 11 times greater among children with SAM.^3^ Given the large burden of MAM and possibility of progression to more life-threatening conditions such as SAM without adequate support, there is increasing recognition that the management of MAM should be considered a public health priority.

In response to concern regarding the limited effectiveness of conventional approaches in the management of MAM usually including a premix of blended food or cereal flour, other dietary supplements have been considered, including the use of ready-to-use foods and improved formulations of fortified blended flours.^4^ Ready-to-use foods have been shown to be highly effective in the treatment of SAM^5–7^ and are increasingly used in the treatment and prevention of MAM,^8–11^ but their high cost raises questions regarding their long-term sustainability given the large global burden of MAM. New formulations of fortified blended flours with improved nutritional profiles may be a less expensive alternative for large-scale programmes, but rigorous scientific evidence to support their use remains limited.^12–14^

In 2010, a cluster-randomised trial was conducted to assess the effectiveness of four dietary supplements in the management of MAM among young children treated at outpatient health facilities in rural Mali.^15^ As part of this trial, we estimated the costs and cost-effectiveness of the four dietary supplements in terms of cost per death averted and per disability-adjusted life years (DALYs) averted.

## Methods

We undertook an incremental cost-effectiveness analysis within a cluster-randomised trial designed to assess the effectiveness of four dietary supplements in the management of MAM. The trial was conducted from May 2010 to August 2011 in the Dioila Health District of Mali. Details of the trial design are presented elsewhere.^15^ In brief, 12 community health centres were randomised to provide one of four dietary supplements containing 500 kcal/day: (1) ready-to-use supplementary foods (RUSFs, PlumpySup; Nutriset, France): a ready-to-use soy protein, peanut paste enriched with a vitamin–mineral complex; (2) CSB++: a specially formulated corn–soy blend containing dehulled soybean flour, maize flour, dried skimmed milk, soy oil and a micronutrient pre-mix (Super CerealPlus, Michiels Fabrieken, Belgium); (3) Misola (MI): a locally produced, micronutrient-fortified, cereal-legume blend containing millet or maize, soy and peanut flour (Misola, Mali); or (4) locally milled flour mixture (LMF): a mixture of home-available foods, including millet, beans, oil and sugar, as is currently recommended by the national treatment protocol for acute malnutrition^16^ when specially processed foods are not available, as well as a 1 g sachet of a multiple micronutrient powder (MixMe; DSM, South Africa) to be added after cooking.

Study villages (n= 95) received community-based screening for acute malnutrition (MAM or SAM) every 2 months. Children 6–35 months of age and identified with MAM during screening, defined in the trial using either the 2006 WHO growth standards (−3≤weight for length Z-score [WLZ] <−2 or 11.5 cm≤mid upper arm circumference [MUAC] <12.5 cm) or the national protocol at the time of the study^16^ (70%≤National Center for Health Statistics weight-for-length median<80% or 11 cm≤MUAC<12 cm), were referred to the nearest community health centre where study eligibility was confirmed.

All children received standard care according to the national protocol for the community-based management of MAM. Systematic treatment, including high-dose vitamin A capsule and antihelminthic treatment, were provided on admission, as well as any specific treatment required for malaria or other acute infections. The dietary supplements were distributed during clinic visits scheduled on a weekly basis for the first 4 weeks and biweekly thereafter for 12 weeks. At each scheduled visit, children received a physical examination and anthropometric assessment using standard techniques. Children found to have developed SAM (defined as MUAC <11.5 cm or WLZ <−3) or requiring inpatient care were referred to the nearest therapeutic feeding programme (TFP) or hospital, respectively, for treatment.

### Costs and costing assumptions

Costs were assessed from the perspective of the healthcare provider. An ingredients approach^17^ was used to identify and cost all resources required for the two principal activities in the community-based management of MAM: bimonthly community-based screening and MAM treatment. Each activity was composed of four cost categories: personnel; supplementary food; medical supplies and materials; infrastructure and logistical support. For each category, we created a cost inventory and quantified resources used throughout the course of treatment.

For the community-based screening activity, we calculated the costs of screening per child as the cost of conducting bimonthly community-based screening for 1 year divided by the number of children with SAM or MAM detected in the same period, to avoid issues of seasonality in case detection. For the MAM treatment activity, total treatment costs were calculated as the sum of infrastructure costs per child and recurrent costs (including personnel, supplementary food, and medical supplies and materials) per enrolment or follow-up visit, where recurrent costs per follow-up visit were multiplied by the number of follow-up visits by arm. The analysis considered supplementation for MAM treatment to continue until the child experienced one of five MAM treatment outcomes: recovery, defined as attaining WLZ >−2.0 and MUAC >12.5 cm on at least two consecutive follow-up visits; default, defined as missing two consecutive follow-up visits; non-response, defined as not meeting the criteria for recovery at 12 weeks; development of SAM and transfer to TFP or hospital; death.

Costs were estimated for a standard programme based on review of financial documents from the trial and key informant interviews at the Dioila Health District. These interviews were used to elicit estimates of resource use required if the programme for management of MAM was implemented by government counterparts. All costing assumptions are presented in detail in online supplementary appendix 1 and table 1.

10.1136/bmjgh-2018-001227.supp1 Supplementary data

**Table 1:** Costing analysis of community-based screening and treatment of acute malnutrition in Mali

Activity	Cost per child (2015 USD)	% of activity total	Comments
1. Community-based screening			
Personnel	1.46	77%	Includes stipend for 2 community volunteers, 1 day per village per screening
Infrastructure and logistical support	0.26	14%	Includes basic furniture (table and chairs) and MUAC bands
Management and administration	0.17	9%	10% of direct costs^29^
Total*	1.89	100%	1766 children were identified with MAM or SAM
2. MAM treatment†			
Personnel	8.30	22%–30%	Includes 1 nurse (20 min) and 1 nurse assistant (10 min); US$1.66 per visit
Infrastructure	6.46	17%–23%	Includes semipermanent building with 1 storekeeper, 1 guard and 1 cleaner, anthropometric equipment (scale, height board and MUAC bands), furniture (consultation table, desk, chairs, benches and water container), medical equipment for physical examination (stethoscope, thermometer and otoscope), enrolment register, equipment and printed communication tools for cooking demonstrations
Medical supplies and materials	2.67	7%–10%	Includes vitamin A, deworming tablet, iron-folic acid, rapid malaria test, malaria treatment, and beneficiary card at enrolment and a disposable tongue depressor at each visit
Supplementary foods			
RUSF	17.25	45%	US$3.45 per weekly ration, includes food costs and domestic and international transport
CSB++	8.10	29%	US$1.62 per weekly ration, includes food costs and domestic and international transport
Misola	7.85	28%	US$1.57 per weekly ration, includes food costs and domestic and international transport
LMF	8.50	30%	US$1.70 per weekly ration, includes food costs and domestic and international transport
Management and administration	2.52–3.46	9%	10% of direct costs^29^
Total	27.76–38.10	100%	
3. SAM treatment			
Direct cost per child treated	120.33	73%	Includes personnel, therapeutic food, medical supplies and materials, infrastructure and logistic support for outpatient care and inpatient care^30^
Management and administration	44.79	27%	37% of direct costs^30^
Total	165.12	100%	

*When considering SAM treatment only, community-based screening would cost 14.51 per SAM child identified, including US$11.19 for personnel, US$2.00 for infrastructure and logistical support, and US$1.32 for management and administration with 231 children identified with SAM.

†Total costs of MAM treatment were calculated for 5 weeks of follow-up because the mean time to recovery was between 4 and 5 weeks for all four dietary strategies (table 2).

CSB, corn–soy blend; LMF, locally milled flour; MAM, moderate acute malnutrition; MUAC, mid upper arm circumference; RUSF, ready-to-use supplementary food; SAM, severe acute malnutrition.

**Table 2:** Parameter values for decision tree model

Parameter	Base Case	Distribution	Range for one-way sensitivity analysis
**Natural history**			
Annual background mortality rate for non-wasted children 1-5y in Mali^23 31^	1.7%	Beta: α=114, β=6556	1.4–2.0%
DALY lost due to death from MAM or SAM^32^	27.8	Fixed	19–60
Proportion of malnutrition cases with SAM^15^	13.1%	Beta: α=3, β=20^*^	2.9–29%
Probability of developing SAM among children with MAM^33^	9.3%	Beta: α=4, β=39*	3.0–19.5%
HR of mortality among children with untreated MAM^3^	3.4	Log-normal: μ=1.2, σ=0.09	2.8–4.0%
HR of mortality among children with untreated SAM^3^	11.6	Log-normal: μ=2.45, σ=0.09	9.7–13.8%
Duration of untreated SAM episode (weeks)^34^	20.2	Log-normal: μ=2.98, σ=0.22	12.8–30.3
Duration of untreated MAM episode (weeks)^34^	11.6	Log-normal: μ=2.45, σ=0.08	10.0–13.4
HR of mortality among children post-recovery^35^	1.2	Log-normal: μ=0.18, σ=0.18*,†	1–1.6
**SAM treatment**			
Duration of SAM treatment (weeks)^36^	6.3	Log-normal: 1.8, 0.3	3.0–11.0
Probability of defaulting from SAM treatment programme^37^	8.0%	Beta:α=4, β=46*	2.3–16.9%
Weight for calculating average of the duration of SAM and MAM among defaulters (higher weight assumes defaulters are more like recovered children; lower weight assumes defaulters are more like untreated children)	50%	Beta: α=1, β=1*	2.5–97.5%
**MAM treatment arm in parent trial**			
**RUSF Treatment**			
Probability of recovering from MAM after RUSF treatment	69.9%	Beta: α=234, β=101	64.8–74.5%
Probability of defaulting from RUSF treatment	6.6%	Beta: α=22, β=313	4.2–9.5%
Average Weeks to recovery	4.3	Weibull: shape=1.3, scale=4.6	0.3–12.9
Average Weeks to default	5.7	Weibull: shape=2.2, scale=6.5	1.2–12.0
**CSB++ Treatment**			
Probability of recovering from MAM after CSB++treatment	61.1%	Beta: α=209, β=133	55.9–66.2%
Probability of defaulting from CSB++treatment	4.1%	Beta: α=14, β=328	2.3–6.4%
Average Weeks to recovery	4.2	Weibull: shape=1.2, scale=4.4	0.2–13.1
Average Weeks to default	5.0	Weibull: shape=1.8, scale=5.6	0.7–11.9
**MI Treatment**			
Probability of recovering from MAM after MI treatment	57.2%	Beta: α=175, β=131	51.6–62.6%
Probability of defaulting from MI treatment	7.8%	Beta: α=24, β=282	5.1–11.1%
Average Weeks to recovery	4.7	Weibull: shape=1.2, scale=5.0	0.3–14.6
Average Weeks to default	4.0	Weibull: shape=1.4, scale=4.4	0.3–11.3
**LMF Treatment**			
Probability of recovering from MAM after LMF treatment	57.7%	Beta: α=162, β=119	51.8–63.4%
Probability of defaulting from LMF treatment	1.1%	Beta: α=3, β=281	0.2–2.9%
Average Weeks to recovery	4.8	Weibull: shape=1.2, scale=5.1	0.2–15.6
Average Weeks to default	9.4	Weibull: shape=7.84, scale=9.9	6.3–11.7
**Adverse Events**			
Probability of incident SAM/hospitalisation during MAM treatment	0.4%	Beta: α=5, β=1264	0.1–0.8%
Average weeks to hospitalisation or SAM	6.3	Weibull: shape 1.2, sigma 6.6	0.3–19.7

*Distribution set so that SD is 50% of the mean such that the 95% interval will be approximately ±100% of the mean.

†Hazard ratios were set to equal the maximum of one or the random draw from the log-normal distribution such that the post-recovery probability of death would not be less than the background mortality.

CSB, corn–soy blend; DALY, disability-adjusted life year; LMF, locally milled flour; MAM, moderate acute malnutrition; MI, Misola; RUSF, ready-to-use supplementary food; SAM, severe acute malnutrition.

### Model structure and transition probabilities

**Figure 1:**
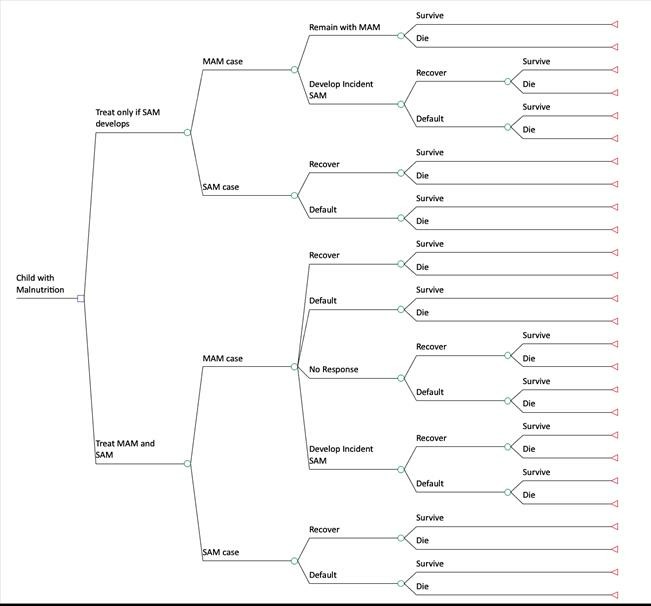
Schematic of decision tree model. MAM, moderate acute malnutrition; SAM, severe acute malnutrition.

We used a decision tree model to compare the four dietary supplements examined in the trial with a hypothetical alternative of providing treatment for SAM only (figure 1). For the four dietary supplements considered for MAM treatment, the probability of each treatment outcome (recovery, default, non-response, transfer to TFP or hospital) was based on the observed outcomes of the 1264 children enrolled in the trial (table 2). As the number of transfers to TFP or hospital observed in the trial was small (n=4), we assumed no group-wise differences in the number of hospitalisations. Transition probabilities not observed in the trial, including outcomes of untreated MAM, were taken from the published literature (table 2).

### Analysis

We used the decision tree model to estimate costs incurred, probability of death and number of DALYs averted (see online supplementary appendix 1). We assumed that individuals alive at the end of 1 year faced the same age-specific mortality risks as the general population. We calculated DALYs averted for one strategy compared with another as the difference in discounted life expectancy achieved by each strategy.

For each treatment pathway, the expected costs and effects were calculated using the probability of each treatment outcome observed in the trial or estimated from the relevant literature. Dominated strategies, which were defined as those that were associated with higher costs and worse outcomes compared with one or a combination of strategies, were identified and removed from consideration. Incremental cost-effectiveness ratios (ICERs) were then calculated among non-dominated strategies as the ratio of expected incremental costs and expected incremental effects for one strategy compared with the next least expensive. ICERs can be compared with external estimates of cost-effectiveness thresholds, with the optimal intervention being the intervention with the highest ICER that is less than the external threshold.^18 19^

Probabilistic sensitivity analysis (PSA) is used to understand how the combined uncertainty in the parameters of an analysis produce uncertainty in final results.^20^ PSA was conducted using probability distributions defined for each model parameter (table 1) generated with 10 000 simulations and assuming all parameters to be uncorrelated.^21^ When distributions for parameters could not be estimated from the trial data or relevant literature, we set the SD for the distribution equal to 50% of the mean, such that an equal-tailed 95% interval would be approximately ±100% of the mean. Cost-effectiveness acceptability curves were plotted to identify the probability that a given strategy was the most cost-effective option over a range of willingness-to-pay values (i.e. the value below which providing a service is deemed cost-effective, equivalent to the cost-effectiveness threshold commonly defined by a country’s per capita Gross Domestic Product [GDP]).^19^ One-way sensitivity analyses were conducted for all key parameters to show the relative influence of single parameters on the estimated ICERs and presented as a tornado diagram. Sensitivity analyses varied each cost parameter by ±50%, the discount rate used in the calculation of the DALY from 0% to 5%, and all other model parameters from the 25th to the 97th percentiles of their probability distribution (table 2). In an additional sensitivity analysis, we considered a ‘do nothing’ scenario in which no screening occurred and neither SAM nor MAM treatment was available. While this hypothetical scenario is inconsistent with WHO guidance that supports the community-based management of acute malnutrition, it is representative of many resource-limited settings. In the ‘do nothing’ scenario, children with untreated SAM could either recover or die. Children with MAM could remain with MAM and either recover spontaneously and survive or die, or progress to SAM. All analyses were conducted using TreeAge Pro 2013 (TreeAge Software, Williamstown, Massachusetts, USA) and STATA V.14.2 (StataCorp, College Station, Texas, USA).

## Results

### Health outcomes

Progression to SAM at 1 year was estimated to be 8.1% without MAM treatment and 0.3% with MAM treatment. The 1-year risk of death for untreated MAM (‘Treat SAM only’) was 3.59% (table 3). Compared to SAM treatment only, MAM treatment with RUSF, CSB++, MI and LMF reduced the risk of death by 0·53 (95% CI: -0·08, -1·03), 0·43 (95% CI: -0·01, -0·92), 0·41 (95% CI: 0·03, -0·88), and 0·35 (95% CI: 0·10, 0·82) percentage points (15·4%, 12·7%, 11·9%, and 10·3%), respectively.

**Table 3:** Incremental outcomes and cost-effectiveness ratios for competing treatment strategies

Strategy	Average cost per child identified (US$)	Probability of death at 1 year for a child presenting with MAM (%)	Discounted life expectancy for a child presenting with MAM (years)	Incremental cost per death averted	Incremental cost per DALY averted
Treat SAM only	36.96	3.42	26.85	Referent	Referent
Treat MAM with RUSF	89.01	2.89	27.00	US$9820.75	US$347.00
Treat MAM with CSB++	90.43	2.99	26.97	Dom.	Dom.
Treat MAM with MI	90.86	3.01	26.96	Dom.	Dom.
Treat MAM with LMF	99.91	3.06	26.95	Dom.	Dom.

Dom. indicates that a strategy was dominated by another strategy (or combination of strategies) being considered.

CSB, corn–soy blend; DALY, disability-adjusted life year; LMF, locally milled flour; MAM, moderate acute malnutrition; MI, Misola; RUSF, ready-to-use supplementary food; SAM, severe acute malnutrition.

### Costs

For the average 5-week course of supplementation observed in the trial, supplementary foods represented 28%–45% of total MAM treatment costs (table 1). Personnel was an important contributor to total MAM treatment costs (22%–30%), while routine medical supplies and materials were less important (7%–10% of total MAM treatment costs). Community-based screening costs constituted only 4.7% of costs for the ‘Treat SAM only’ arm and 1.7%–1.9% costs for the MAM treatment arms.

### Cost-effectiveness

For all cost-effectiveness outcomes, the CSB++, MI and LMF strategies were dominated, for example, they were associated with worse outcomes and higher costs. RUSF was estimated to have an ICER of US$9821 per death averted and US$347 per DALY averted, as compared with the ‘Treat SAM only’ strategy. In a context where RUSF is not available, the ICERs for CSB++, MI and LM compared with ‘Treat SAM only’ were $12,435, $13,146, and $17,486 per death averted and $446, 490, and 630 per DALY averted, respectively.

**Figure 2:**
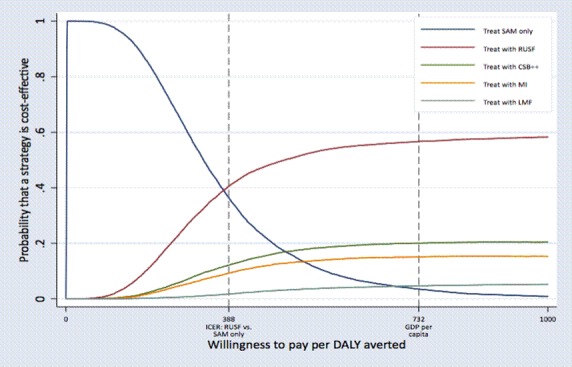
Cost-effectiveness acceptability curves for competing treatment strategies, using DALY averted as the outcome. CSB, corn–soy blend; DALY, disability-adjusted life year; GDP, gross domestic product; ICER, incremental cost-effectiveness ratio; LMF, locally milled flour; MI, Misola; RUSF, ready-to-use supplementary food; SAM, severe acute malnutrition.

The ‘Treat SAM only’ strategy was most likely to be cost-effective up to a willingness-to-pay of US$347 per DALY averted (figure 2). At higher willingness-to-pay values, additional MAM treatment with RUSF was the preferred option and had a 56% probability of being the optimal strategy at a willingness-to-pay of US$732 (equal to the Mali gross domestic product [GDP] per capita in 2015). At this willingness-to-pay value equal to the Mali GDP per capita in 2015, the ‘Treat SAM only’ strategy had just a 4.0% probability of being the optimal strategy. The probability of the CSB++, MI or LMF strategies being the optimal strategy under any willingness-to-pay value was less than 22%, 16% and 6%, respectively.

**Figure 3:**
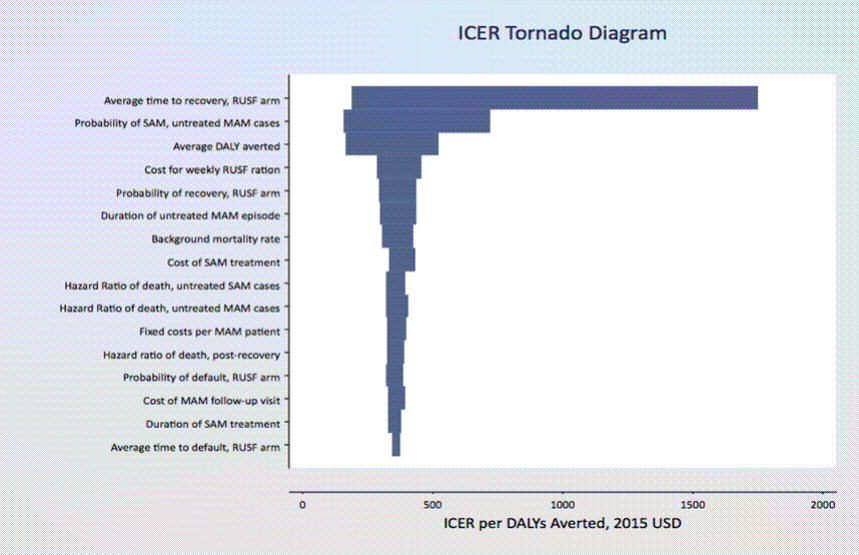
Tornado diagram of one-way sensitivity analyses on key model parameters. Parameters that changed the ICER by less than US$10 were excluded from the figure. DALY, disability-adjusted life year; ICER, incremental cost-effectiveness ratio; MAM, moderate acute malnutrition; RUSF, ready-to-use supplementary food; SAM, moderate acute malnutrition.

### Sensitivity analyses

Average time to recovery in the RUSF arm was a major source of uncertainty; however, compared with the ‘Treat SAM only’ strategy, the ICER for RUSF only exceeded Mali’s GDP per capita if the average recovery time exceeded 8.8 weeks, more than twice the average recovery time observed in the trial (figure 3). The probability of developing SAM among untreated MAM cases was also highly influential; higher probabilities of developing SAM corresponded to more favourable cost-effectiveness ratios for RUSF treatment due to the increased probability of averting incident SAM with RUSF treatment.

**Figure 4:**
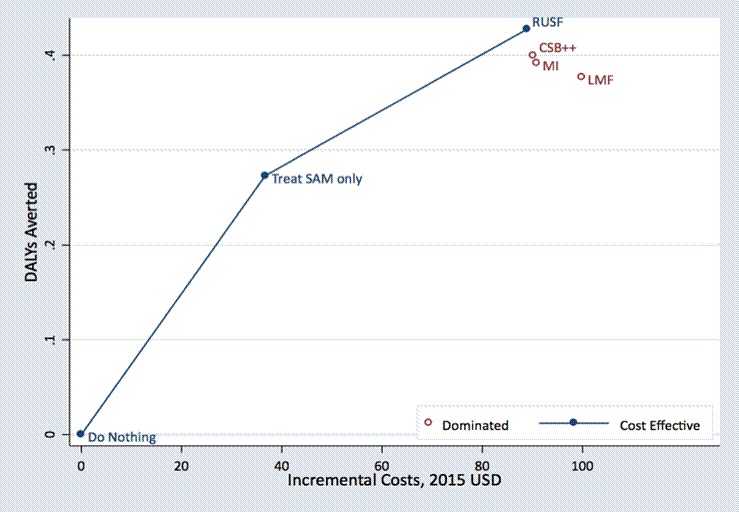
Cost-effectiveness plane (incremental cost per DALY averted) comparing ‘do nothing,’ ‘treat SAM only,’ and 4 MAM treatment strategies. CSB, corn–soy blend; DALY, disability-adjusted life year; LMF, locally milled flour; MI, Misola; RUSF, ready-to-use supplementary food; SAM, severe acute malnutrition.

The decision to provide treatment depended on the willingness-to-pay threshold, with the ‘Treat SAM only’ strategy cost-effective at willingness-to-pay values between US$142 and US$347 per DALY averted and providing RUSF treatment for MAM becoming cost-effective at willingness-to-pay values greater than US$347 (figure 4).

## Discussion

Using primary data from a recent cluster randomised trial combined with estimates from secondary published data, our results show that providing MAM treatment is cost-effective across a wide range of cost-effectiveness thresholds. Despite having the highest per-unit food costs, we found that RUSF was the optimal supplement for MAM treatment when compared with three other supplementary food options.

Several studies have reported the effectiveness of individual dietary supplements for the treatment of MAM,^8 9 12 22^ but our results also provide evidence on the cost-effectiveness of MAM treatment. There is no universal definition of a threshold ratio below which an intervention is considered cost-effective, but WHO has suggested that interventions with cost-effectiveness ratios <1 times per capita GDP per DALY averted for a given country (US$732 in Mali) be considered ‘very cost-effective’ and <3 times per capita GDP per DALY averted (US$2196 in Mali) be considered ‘cost-effective’.^19^ With an estimated cost of US$338 per DALY averted, RUSF treatment for MAM could be considered very cost-effective in this setting. The CSB++, MI and LMF supplements were dominated in this analysis by RUSF, but the ICER for these strategies compared with ‘Treat SAM only’ were favourable and ranged from US$446 to US$630, suggesting that in situations where RUSF were unavailable or there were other reasons to prefer these alternatives, they would also be very cost-effective.

Overall, despite the relatively small gain in absolute survival estimated from our trial and secondary data, community-based MAM treatment has the potential to avert a high number of deaths due to the large burden of MAM. If the mortality reduction estimated in our analysis (0.55% for RUSF) is applied to the population of children under 5 with MAM (330 000 in Mali and 34.1 million globally) then providing MAM treatment in addition to SAM treatment would avert over 1800 deaths in Mali and 187 000 deaths globally each year.^1 23^ MAM treatment also maintains a favourable cost-effectiveness ratio due to its low overall costs. The cost-effectiveness of this intervention compares favourably with other basic health interventions that are generally considered to be cost-effective, such as the provision of oral rehydration solution for the management of diarrhoea (ICER=US$150 per DALY averted), intrapartum care (ICER=US$200–500 per DALY averted) and treating obstructed labour with Caesarean delivery (ICER=US$1600–2600 per DALY averted).^24^ Sensitivity analyses (figure 3) suggest MAM treatment would remain very cost-effective even at reduced effectiveness or greater food costs. Decreasing treatment delivery costs through integration with routine health services or achieving economies of scale with higher coverage and/or more cases treated may further improve the cost-effectiveness of MAM treatment.

Supplementary foods, personnel and infrastructure each contributed an important proportion to total MAM treatment costs, but none materially influenced conclusions regarding the overall cost-effectiveness of MAM treatment. Community-based screening, which has the potential to promote early case finding and increase treatment referrals,^25^ represented a relatively small sum in the management of acute malnutrition. Greater investment in increasing screening frequency or improving screening efficiencies, such as through increased community mobilisation, could increase programme coverage, reduce per-capita fixed costs associated with treatment and prevent the more costly management of complicated cases.

The decision tree model synthesised multiple data sources, and we accounted for uncertainty in each of these data sources using probabilistic sensitivity analysis (tables 1 and 2). Additional one-way sensitivity analysis (figure 3) showed that results were most sensitive to assumptions about average time to recovery in the RUSF arm (4.3 weeks in the parent trial and base case) and progression to SAM without treatment (9.3% in the base case). In our model, longer times to recovery correspond to both increased costs of MAM treatment as well as increased probability of mortality as children remained in the hazardous MAM condition for a longer period. Sensitivity analyses suggest that even assuming a 8.8-week time to recovery, the cost per DALY averted by RUSF treatment would remain highly cost-effective. Data from additional effectiveness studies—which vary the quantity of supplement given, duration of treatment or discharge criteria in varied and programmatic settings—can provide more evidence on the expected clinical effectiveness of dietary supplements and would be valuable to resolve uncertainty for more precise cost-effectiveness estimates. Food costs had relatively little impact on the cost-effectiveness of MAM treatment overall but were influential in the choice between MAM dietary supplements (RUSF vs CSB++, results not shown).

We found providing SAM treatment only to be highly cost-effective (ICER=US$3974 per death averted and US$142 per DALY averted). This finding is consistent with previous reports from African settings suggesting community-based SAM treatment is cost-effective, relative to no SAM treatment. These earlier studies (from 2007 to 2008) reported cost-effectiveness ratios of US$1365–1760 per death averted and US$42–53 per DALY averted,^26 27^ lower than estimated in our study, reflecting differences in price levels and in assumptions regarding the mortality risk of untreated SAM. Our study adds evidence to suggest that expanding services to provide treatment for both MAM and SAM is a cost-effective extension of SAM services at a willingness-to-pay value above US$347 per DALY averted.

This study setting in Mali supported high recovery and low mortality, and is likely to have reduced the observed progression to SAM, hospitalisation and death. The health system provides a basic package of health services, which would be similar to that provided in other resource-constrained settings at the primary care level. Overall, the benefits of MAM treatment in Mali can likely be generalised to similar contexts in sub-Saharan Africa, although contextual factors, such as SAM burden, programme effectiveness and coverage and supplement utilisation within the household, may affect the degree to which the results can be directly extrapolated.

This analysis has several limitations. First, participant recruitment, clinical management and costs in the parent trial may differ from programmatic settings. In this analysis, we assumed clinical outcomes observed in the parent trial could be maintained in programmatic settings. While cost variables were also extrapolated from the trial setting, we were able to omit costs of protocol-driven procedures that would not be relevant in routine programme settings, including the cost of study-specific personnel, the cost of data management and the cost of follow-up after recovery. Further studies conducted in the context of ongoing programmes should be used to inform and update these parameters; effectiveness and food costs may vary across settings and be a source of uncertainty. Second, in building the model, we assumed that differences in health effects and costs would be realised in the 12 months following initial presentation, and that there would be no difference in cost or survival among children alive at the end of this period. It is plausible that children who recover under MAM treatment could be at higher risk of poor future health, related to having experienced MAM, but evidence on long-term consequences is lacking. Third, we did not include household costs associated with MAM treatment. A societal perspective would have allowed for the most inclusive perspective possible, incorporating the potential benefits, harms and costs for all parties involved. Including household costs associated with lost productivity among caregivers due to supplement preparation and clinic visit attendance in this analysis would have likely strengthened the cost-effectiveness of RUSF relative to other dietary supplements, which require cooking and potentially longer duration of treatment. Finally, to make results most relevant to real-world decisions, we focused on a narrow set of dietary supplements. New programmes that harmonise the treatment of MAM and SAM with therapeutic foods at variable doses have been field-tested.^28^ As the absolute resources required to treat MAM would be substantial due to the large burden, strategies that focus on prevention of MAM should also be considered to reduce treatment caseloads and absolute programme costs.

## Conclusion

Cost-effectiveness analysis may be used to inform national and international decision-making and to direct resources towards interventions with the greatest potential to improve child health. MAM is associated with a large proportion of the burden of child morbidity and mortality, but appropriate treatment can both reduce the duration of a MAM episode and the risk of progression to SAM. The potential public health impact of treating MAM is therefore great. Our findings suggest that MAM treatment is a cost-effective extension of existing SAM services across a range of willingness-to-pay thresholds and has the potential to be a promising public health investment in settings with available resources.
