# Supplementary material for: Cost-effectiveness of community-based screening and treatment of moderate acute malnutrition in Mali
Source: BMJ Glob Health. 2019 Apr 28;4(2):e001227. doi: 10.1136/bmjgh-2018-001227 (PMC6509694; doi:10.1136/bmjgh-2018-001227)

**Supplementary Material**

Supplementary Methods Appendix

**Cost-effectiveness analysis**. Cost-effectiveness analysis is a quantitative approach for comparing health interventions, judging each intervention according to its incremental cost and health outcomes relative to the other interventions being considered^1^. Health outcomes can include the number of cases treated, lives saved, and Disability-Adjusted Life Years (DALYs) averted by the intervention. Costs can include the resources required to deliver the intervention, as well as other costs or cost savings attributable to the intervention. The cost-effectiveness of an intervention is described by the incremental cost-effectiveness ratio (ICER), which is calculated by dividing the incremental costs of an intervention by the incremental health benefits, as compared to an alternative intervention. For example, in the main analysis the incremental cost per death averted for ‘Treat MAM with RUSF’, as compared to the ‘Treat SAM only’ strategy, is calculated as the incremental cost ($89·01 - $36·96 = $52.05) divided by the incremental reduction in mortality risk (0.0342 – 0.0289 =0.0053). This ratio (52.05/0.0053 = 9,821) is the ICER for ‘Treat MAM with RUSF’, as compared to the ‘Treat SAM only’ strategy.

This ICER can be compared to other uses of healthcare resources, with this opportunity cost operationalized as a willingness-to-pay-threshold (WTP). Interventions with an ICER below this threshold generate more health gains per dollar than other uses of funding. Cost-effectiveness analysis can be used to guide policy and practice decision-makers in the selection of interventions that produce the greatest health benefits for a given budget. These analyses should be complemented with additional contextual information, such as the burden of disease, existing coverage of health interventions, and capacity of the health system, in final decision-making.

**Costs and costing assumptions**. Resource use in the context of a research study may not reflect resource use in programmatic settings. We therefore present costs estimated for a standard program based on review of financial documents from the parent trial and key informant interviews with clinical and finance officials at the Dioila Health District. These interviews were used to elicit estimates of resource use required if the program for management of MAM was implemented by government counterparts. All capital items were annualized using a 3% discount rate. Useful years of life were assumed to be 1 year for all items valued at less than 100 USD, 2 years for all cooking equipment and furniture, and 5 years for all other items. Program management costs were not directly assessed in the parent trial but assumed to be 10% ^2^. Costs were expressed in local CFA whenever possible and converted to 2015 US Dollars using market exchange rates ^3^. The costs of therapeutic feeding for the treatment of SAM were taken from a recent costing analysis of SAM treatment conducted in the neighboring country of Niger ^4^, and included the cost of inpatient and outpatient care, as well as management and administration.

**Model structure***.* We developed a decision tree model describing the possible pathways in the clinical management of a child presenting with acute malnutrition at screening. We modeled outcomes for one year following identification of MAM or SAM, assuming all differences in resource utilization, morbidity, and mortality would be realized by the end of this year. For those alive at the end of the year we calculated remaining life expectancy using standard life tables. The tree begins with the decision to treat only children who have SAM, or treat children with SAM or MAM using 1 of the 4 dietary supplements from the parent trial described above. In each arm, children identified with SAM initiate treatment and subsequently die, default, or recover, as described below in more detail. In the scenario where no MAM treatment is provided, children with MAM at screening can either remain with MAM and either recover spontaneously and survive or die, or be treated for incident SAM after progression. Where MAM treatment is provided, children with MAM at screening can experience one of 4 MAM treatment outcomes (e.g. recovery, default, non-response and transfer to TFP or hospital, as defined above). Within each of these 4 MAM treatment outcomes, individuals are followed until death or survival 12 months later.

**Transition probabilities***.*  For the 4 dietary supplements considered for MAM treatment, the probability of each treatment outcome (recovery, default, non-response, transfer to TFP or hospital) was based on the observed outcomes of the 1264 children enrolled in the parent trial (Table 1). As the number of transfers to TFP or hospital observed in the parent trial was small (n=4), we assumed no group-wise differences in the number of hospitalizations.

There was no ‘Treat SAM only’ arm in the parent trial, so we created this scenario using published evidence on the outcomes of untreated MAM (Table 2). The probability of progressing from MAM to SAM in the absence of treatment was not known, but conservatively assumed to be 9.3% based on recent evidence from Ethiopia where a more specific definition of SAM was applied ^5^. Similarly, the probability of default among children receiving SAM treatment was not known but was assumed to be 8%, which reflects the median probability of defaulting and death based on a review of over 60 community-based SAM treatment programs implemented by NGOs between 2000 and 2006 ^6^.

For each combination of treatment outcomes, the probability of death over a 12-month period was calculated as a function of the baseline mortality rate among non-wasted children in Mali; the reported hazard ratios of death among children with MAM (hazard ratio (HR)=3.4) and SAM (HR=11.6) ^7^ and after recovery from SAM/MAM (HR=1.2) relative to non-wasted children ^8^; and the duration of time spent in MAM, SAM, and post-recovery health states during the 12-month period. The baseline mortality rate was calculated by using the probabilities of death among children aged 0-1 years and 1-5 years in Mali ^9^, removing deaths attributable to wasting based on estimates from the Global Burden of Disease Study ^10^, and taking a weighted average of these two probabilities based on the age distribution of our target population (6-35 month olds). Treatment was assumed to affect health status by shortening the duration of time spent in the highly-hazardous health states of SAM and MAM. For each MAM treatment strategy, the duration of treated MAM episodes among those who recovered, did not respond, or developed SAM was calculated using data from the main trial. The duration of untreated MAM and SAM episodes, and treated SAM episodes was assumed to be 11.6 weeks, 20.2 and 6.3 weeks, respectively ^11,12^. For defaulters, the duration of a MAM and SAM episode was calculated as an average of the duration of treated and untreated MAM or SAM. For illustrative purposes, Supplemental Figure 1 shows the assumed duration of time spent under various health states and the calculated probability of death for each treatment outcome of the ‘Treat SAM only’ and the RUSF treatment strategies.

**Uncertainty analysis**. To assess uncertainty in model results, we used probabilistic sensitivity analysis (PSA), following published recommendations for model-based cost-effectiveness analysis.^13^ To implement the PSA, we specified probability distributions for each uncertain model parameter (Table 1, ‘Distribution’). These distributions quantify current uncertainty in each parameter going into the model. We then drew multiple samples from the full set of model parameters. Each of these ‘parameter sets’ was use to generate a single realization of model outcomes. We ran the model a large number of times, each time with a randomly drawn set of model parameters, producing a distribution of model results. These results were used to produce uncertainty intervals (calculated as an equal-tailed 95% interval) to describe uncertainty in model outcomes. Intervals calculated for ratio statistics (such as the ICER) can perform poorly when both numerator and denominator can include negative values, and for this reason we use cost-effectiveness acceptability curves (CEACs)^14^ to describe uncertainty in cost-effectiveness results

**References**

1. Drummond MF, Sculpher MJ, Claxton K, Stoddart GL, Torrance GW. Methods for the economic evaluation of health care programmes. Oxford University Press; 2015.

2. Johns B, Baltussen R, Hutubessy R. Programme costs in the economic evaluation of health interventions. Cost Eff Resour Alloc. Feb 26 2003;1(1):1.

3. "Historical Exchange Rates". In: OANDA, ed.

4. Isanaka S, Menzies NA, Sayyad J, Ayoola M, Grais RF, Doyon S. Cost analysis of the treatment of severe acute malnutrition in West Africa. Matern Child Nutr. Oct 2017;13(4).

5. Valid International, Jimma University, Save the Children, Empowering New Generations to Improve Nutrition and Economic Opportunities (ENGINE). Outcomes of Moderate Acute Malnutrition and their determinants in under-five children: a prospective cohort study from a food-secure setting in rural Ethiopia. USAID Agreement No. AID-663-A-11-00017: United States Agency for International Development (USAID) 2015.

6. Guerrero S, Rogers E. Access for All, Volume 1: Is community-based treatment of severe acute malnutrition (SAM) at scale capable of meeting global needs? London: Coverage Monitoring Network; 2013.

7. Black RE, Victora CG, Walker SP, et al. Maternal and child undernutrition and overweight in low-income and middle-income countries. The Lancet. 2013;382(9890):427-451.

8. Bahwere P. Long term mortality after community and facility based treatment of severe acute malnutrition: Analysis of data from Bangladesh, Kenya, Malawi and Niger. Journal of Public Health and Epidemiology. 2012;4(8):215-225.

9. United Nations, Department of Economic and Social Affairs, Population Division. World Population Prospects: The 2015 Revision. 2015.

10. Global Burden of Disease Collaborative Network. Global Burden of Disease Study 2016 (GBD 2016) Results. 2017; <http://ghdx.healthdata.org/gbd-results-tool>.

11. Isanaka S, Grais RF, Briend A, Checchi F. Estimates of the duration of untreated acute malnutrition in children from Niger. Am J Epidemiol. Apr 15 2011;173(8):932-940.

12. Ale BM. Predictive factors of time-to-recovery in children with severe acute malnutrition treated in rural area in Mali: an application of Cox regression. Nairobi: Institute of Tropical & Infectious Diseases, University of Nairobi; 2016.

13. Briggs AH, Weinstein MC, Fenwick EA, et al. Modeling Good Research Practices Task Force. Model parameter estimation and uncertainty: a report of the ISPOR-SMDM Modeling Good Research Practices Task Force-6. Value in Health. 2012;15(6):835-842.

14. Fenwick E, Claxton K, M S. Representing uncertainty: the role of cost‐effectiveness acceptability curves. Health economics. 2001;10(8):779-787.

Supplemental Figure 1. Length of stay in various health states and corresponding annual probability of death


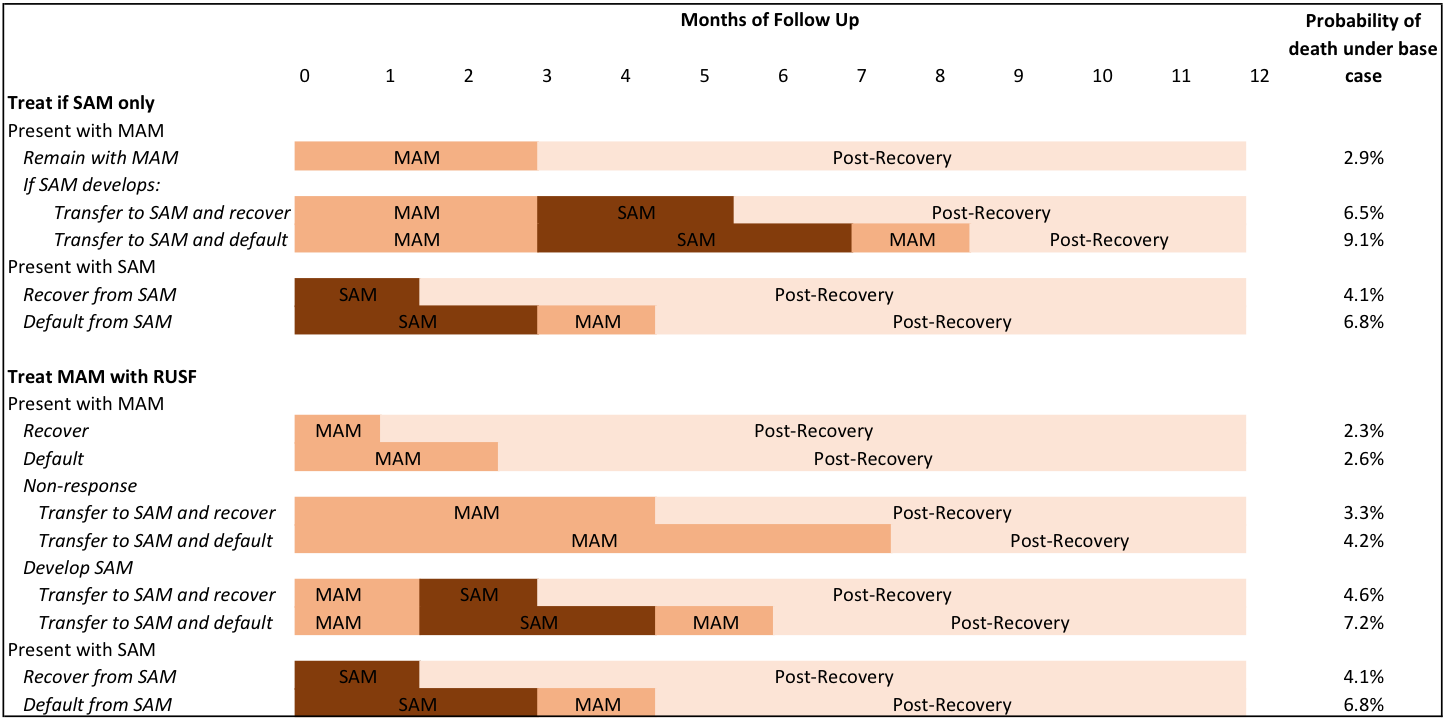

Supplement: Supplementary data [file bmjgh-2018-001227supp001.docx]
